# Antifungal stewardship in practice: Insights from a prospective audit and feedback program

**DOI:** 10.1017/ice.2023.129

**Published:** 2023-12

**Authors:** Laura L. Bio, Yingjie Weng, Hayden T. Schwenk

**Affiliations:** 1 Department of Pharmacy, Lucile Packard Children’s Hospital Stanford, Stanford, California; 2 Quantitative Sciences Unit, Stanford University School of Medicine, Stanford, California; 3 Department of Pediatrics, Stanford University School of Medicine, Stanford, California

## Abstract

**Objective:**

To identify characteristics of antifungal prospective audit and feedback (PAF) and to compare rates of PAF recommendation and acceptance between antifungal and antibiotic agents.

**Design:**

Retrospective cohort study of antifungal and antibiotic audits by a children’s hospital antimicrobial stewardship program (ASP) from November 1, 2020, to October 31, 2022.

**Methods:**

Antimicrobial audit data were retrieved from the ASP data warehouse. We characterized antifungal PAF using descriptive statistics. We then compared the overall rates of PAF recommendation and recommendation acceptance between antifungals and antibiotics. We also compared the differences in antifungal and antibiotic PAF recommendation and acceptance rates across various factors, including infectious problem, medical service, and recommendation type.

**Results:**

Of 10,402 antimicrobial audits identified during the study period, 8,599 (83%) were for antibiotics and 1,803 (17%) were for antifungals. The highest antifungal recommendation rates were for liposomal amphotericin B, antifungals used for sepsis or respiratory tract infection, and antifungals prescribed in the cardiovascular intensive care unit. The rate of PAF recommendation was higher for antibiotics than for antifungals (29% vs 21%; *P* < .001); however, the rates of recommendation acceptance were similar. Recommendations to discontinue or for medication monitoring were more common for antifungals.

**Conclusions:**

Our analysis of antifungal PAF identified key opportunities to improve antifungal use, including the optimized use of specific agents and targeted use by certain medical services. Moreover, antifungal PAF, despite identifying fewer recommendations compared to antibiotic PAF, were associated with similarly high rates of acceptance, highlighting a promising opportunity for antifungal stewardship.

Antifungal medication use in children is increasing, and a significant proportion of this use is inappropriate.^1,2^ Suboptimal antifungal use is associated with worse clinical outcomes, more adverse drug events, and higher costs.^3^ Overprescribing of antifungals contributes to the emergence of antifungal-resistant pathogens, including *Candida auris* and azole-resistant *Aspergillus* spp. In response, the Centers for Disease Control and Prevention and other experts have recommended that hospitals leverage existing antimicrobial stewardship programs (ASPs) to assess antifungal use and the appropriateness of prescribing.^2,4^

The optimal approach to antifungal stewardship remains uncertain and may differ from traditional antibiotic stewardship strategies. A variety of approaches to antifungal stewardship have been evaluated, including prospective audit and feedback (PAF), preauthorization, and utilization of novel fungal diagnostics.^5^ PAF of antifungal prescriptions within hospitals has been shown to improve the quality of prescribing and has been recommended as an essential part of antifungal stewardship.^2,6,7^ Antifungal PAF also provides ASPs with a better understanding of antifungal prescribing patterns at their institution and allows for the identification of suboptimal practices that may benefit from alternative stewardship strategies (eg, institutional guidelines).

In a recent survey of pediatric ASPs, 63% performed antifungal PAF.^1^ However, nearly 20% of hospitals reported performing antifungal PAF <5 days per week, and the antifungal characteristics monitored as part of PAF varied across institutions. Data pertaining to the operationalization of antifungal PAF in pediatric settings are limited, and the characteristics have not been well described. We have described antifungal PAF characteristics at a freestanding children’s hospital with a large immunocompromised patient population. We have highlighted the unique attributes and areas of focus for antifungal PAF by comparing the rates of recommendation and recommendation acceptance between antifungal and antibiotic audits.

## Methods

### Study design

Audits performed on antifungals and antibiotics between November 1, 2020, and October 31, 2022, were included in this analysis. During this time, institution-specific antifungal guidelines included fluconazole prophylaxis in the neonatal intensive care unit (NICU), empiric antifungals for prolonged febrile neutropenia, and antifungal prophylaxis after liver transplant.^8^ The use of posaconazole, isavuconazole, or echinocandins other than caspofungin was restricted and required approval by the pediatric infectious diseases (ID) team.

At our hospital, an ASP pharmacist performs PAF Monday through Friday for all inpatient antimicrobial orders active ≥48 hours, including restricted agents. The ASP pharmacist documents all audits and recommendations in a custom electronic health record-embedded smart form.^9^ Audit characteristics, including antimicrobial name, infectious problem (Supplementary Table 1 online), medical service (Supplementary Table 2 online), and recommendation type (Supplementary Table 3 online), if applicable, were collected for data summary and analysis.

### Statistical analyses

Descriptive statistics were used to characterize antifungal and antibiotic PAF. We compared the overall PAF recommendation and acceptance rates between antifungals and antibiotics with the χ^2^ test using SPSS Statistics version 24 software (IBM, Armonk, NY). To identify predictors of PAF recommendation and acceptance for antifungal PAF compared to antibiotic PAF, we created logistic regression models to evaluate the likelihood of identifying a recommendation based on antimicrobial category (ie, antifungal, antibiotic), infectious problem, medical service, and subsequently, the likelihood of recommendation acceptance based on these categories. We used R Studio version 4.0.3 software (R Foundation for Statistical Computing, Vienna, Austria) for this analysis. First, a logistic regression model was performed by regressing the binary indicator of antimicrobial type (ie, antifungal, antibiotic), the categorical indicator of the infectious problem, and an interaction term regarding whether a recommendation was made. To determine whether the difference in recommendation rates for antifungal and antibiotic audits was homogeneous across infectious problems, we reported marginal mean differences of the recommendation rates from the model with their corresponding 95% confidence intervals (CIs). Second, a similar analysis was conducted to determine whether there was a difference in the proportion of recommendations between antifungal and antibiotic audits by medical service. Third, the difference in acceptance rates between antifungal and antibiotic recommendations was assessed using similar models. Finally, to explore the differences in the proportions of each recommendation type between antifungal and antibiotic recommendations, we assumed a binomial distribution and calculated the corresponding 95% CIs.

## Results

In total, 10,402 audits were performed between November 1, 2020, and October 31, 2022, including 1,803 antifungal and 8,599 antibiotic audits. Of 1,803 antifungal audits, 379 (21%) resulted in a recommendation compared to 2,467 (29%) of 8,599 antibiotic audits (*P* < .001). PAF recommendation acceptance was similar between antifungals and antibiotics: 298 (79%) of 379 and 1,982 (80%) of 2,467, respectively (*P* = .48).

**Figure 1.**
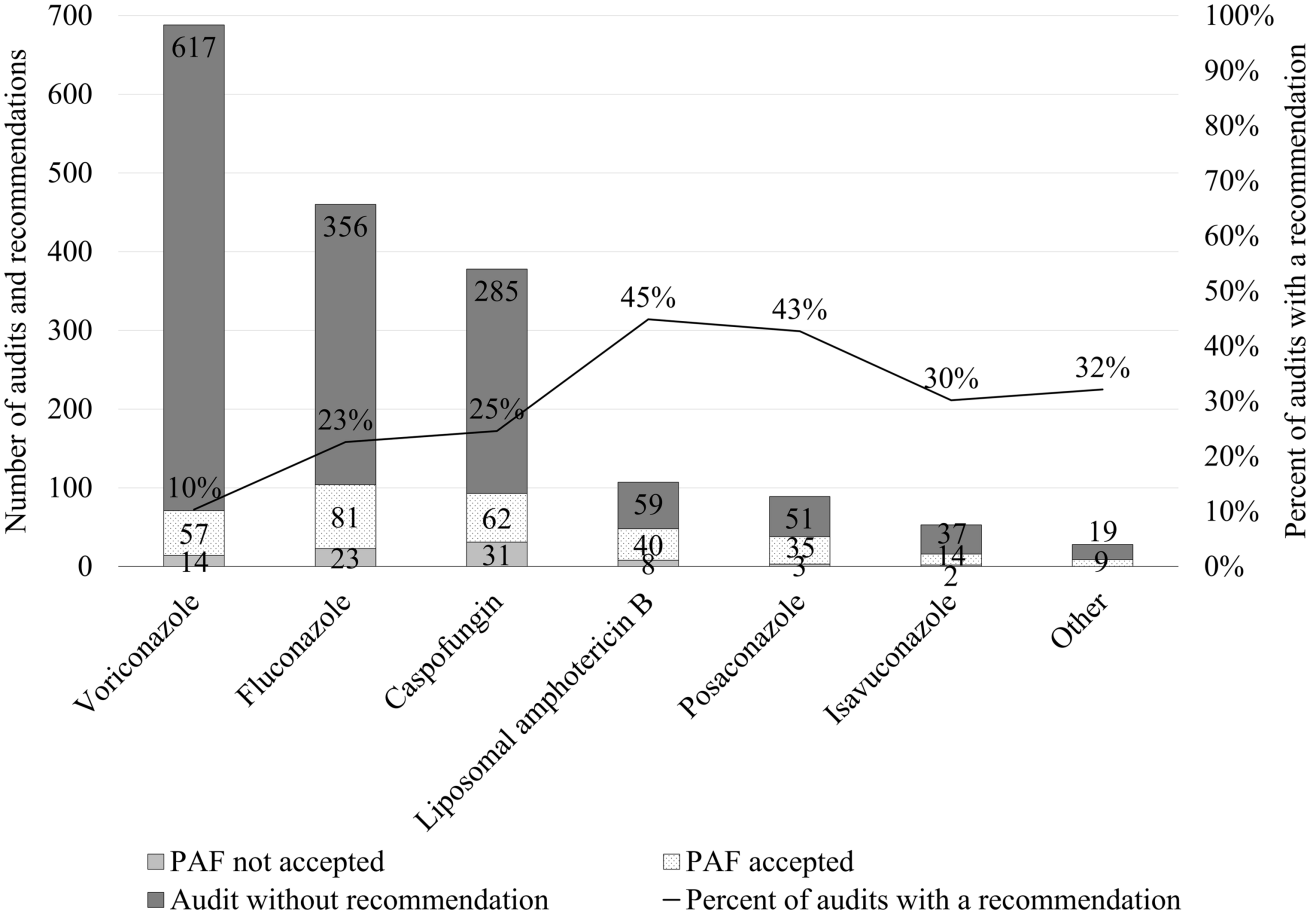
Prospective audit and feedback recommendations of antifungals. Note. PAF, Prospective audit and feedback. Other includes audits for the following antifungals (number): itraconazole (n = 14), nystatin (n = 5), micafungin (n = 3), amphotericin B deoxycholate (n = 2), clotrimazole (n = 2), griseofulvin (n = 1), and terbinafine (n = 1).

The most common antifungals audited were voriconazole and fluconazole (Fig. 1). Liposomal amphotericin B had the highest PAF recommendation rate (45%, 48 of 107), with most recommendations accepted (40 of 48, 83%). Prophylaxis was the most common indication for antifungal use based on PAF volume but had the lowest recommendation rate (10%, 106 of 1,082) (Table 1). Antifungals audited for sepsis or respiratory tract infection had the highest rates of PAF recommendation: 48% (68 of 143) and 47% (78 of 167), respectively. The hematology and oncology unit, combined with the stem-cell transplant unit (Heme/Onc/SCT), had the highest volume of antifungal audits but had a low recommendation rate: 12% (121 of 1,045,). Most recommendations in these units were for patients receiving antifungals for prophylaxis or a respiratory tract infection, and these antifungal indications had markedly different recommendation rates: 5% (38 of 799) and 42% (32 of 77), respectively. Although the cardiovascular intensive care unit (CVICU) had the fourth-highest volume of antifungal audits, it was the unit with the highest antifungal PAF recommendation rate: 47% (100 of 211). Most antifungal recommendations in the CVICU were for sepsis (40 of 80, 50%) followed by prophylaxis (28 of 79, 35%). Overall, the most common antifungal recommendation was to discontinue the agent (119 of 379, 31%), although only 71% (84 of 119) of these recommendations were accepted. The antifungal PAF recommendation that was least likely to be followed was conversion from an intravenous to enteral route of administration (25 of 45, 56%).

**Table 1.** Comparison of Antifungal and Antibiotic Audit, Recommendation, and Recommendation Acceptance Characteristics

	Antifungals, No. (%)			Antibiotics, No. (%)			Adjusted Difference^a^ (Antifungal–Antibiotics)	
Variable	Audits (N=1,803)	Recs (N=379)	Recs Accepted (N=298)	Audits (N=8,599)	Recs (N=2,467)	Recs Accepted (N=1,982)	Recommendation Rate Difference, % (95% CI)	Recommendation Acceptance Rate Difference, % (95% CI)
**Infectious problem (%)**								
Prophylaxis	1,082 (60)	106 (28)	72 (24)	2,211 (26)	253 (10)	180 (9)	0.4 (−4.8 to 5.7)	−5.9 (−25.6 to 13.9)
Respiratory infection	167 (9)	78 (21)	65 (22)	1,238 (14)	452 (18)	365 (18)	**16.7 (1.8 to 31.5)**	−0.4 (−18.9 to 18.1)
Sepsis	143 (8)	68 (18)	46 (15)	1,170 (14)	503 (20)	388 (20)	3.4 (−12.4 to 19.1)	-6.6 (−27.2 to 14)
Head and neck infection	79 (4)	27 (7)	25 (8)	169 (2)	58 (2)	52 (3)	4 (−20.7 to 28.6)	1.2 (−24.7 to 27)
Bloodstream infection	70 (4)	28 (7)	25 (8)	627 (7)	240 (10)	214 (11)	3.1 (−19.5 to 25.7)	1 (−20.3 to 22.4)
Non-infectious	68 (4)	0 (0)	0 (0)	447 (5)	43 (2)	31 (2)	**−10.4 (−15.9 to −5)**	NA
SSTI	64 (4)	14 (4)	14 (5)	631 (7)	231 (9)	183 (9)	−9.7 (−32.8 to 13.4)	**20.3 (10.6 to 30.1)**
Gastrointestinal/IAI	30 (2)	11 (3)	7 (2)	953 (11)	219 (9)	172 (9)	12.2 (−19.6 to 43.9)	−14 (−66 to 38.1)
Febrile neutropenia	28 (2)	8 (2)	6 (2)	125 (1)	39 (2)	28 (1)	−2.5 (−41.0 to 35.9)	3.5 (−64.7 to 71.6)
CNS infection	24 (1)	12 (3)	12 (4)	226 (3)	83 (3)	75 (4)	NA	NA
Urinary tract infection	11 (1)	6 (2)	6 (2)	405 (5)	166 (7)	135 (7)	10.4 (−43.8 to 64.5)	**18.8 (7.7 to 30)**
Bone and joint infection	3 (0	0 (0)	0 (0)	117 (1)	49 (2)	42 (2)	NA	NA
Other	34 (2)	21 (6)	20 (7)	280 (3)	131 (5)	117 (6)	NA	NA
**Medical service (%)**								
Heme/Onc/SCT	1,045 (58)	121 (32)	103 (35)	1,725 (20)	286 (12)	236 (12)	−2.8 (−9.6 to 4.1)	2.1 (−10.6 to 14.8)
Intensive care	267 (15)	95 (25)	78 (26)	1,372 (16)	453 (18)	383 (19)	9.6 (−1.5 to 20.8)	−1.8 (−15.9 to 12.4)
Cardiovascular intensive care	211 (12)	100 (26)	70 (23)	1,437 (17)	509 (21)	375 (19)	**15 (3 to 27.1)**	−1.2 (−17 to 14.6)
Medical services	165 (9)	38 (10)	28 (9)	2,153 (25)	610 (25)	500 (25)	−1.9 (−14.9 to 11)	−9.3 (−35 to 16.5)
Solid-organ transplant	66 (4)	4 (1)	2 (1)	216 (3)	63 (3)	51 (3)	−18.2 (−48.3 to 11.9)	−18.3 (−94.8 to 58.1)
Neonatology	38 (2)	16 (4)	14 (5)	873 (10)	312 (13)	264 (13)	15.5 (−11.9 to 42.8)	3.9 (−24.7 to 32.4)
Surgical services	11 (1)	5 (1)	3 (1)	823 (10)	234 (9)	173 (9)	17.7 (−31.5 to 66.9)	−5.8 (−69.2 to 57.6)

Note. CNS, central nervous system; IAI, intra-abdominal infection; NA, not applicable; SSTI, skin and soft-tissue infection; UTI, urinary tract infection; Heme/Onc/SCT, hematology, oncology, stem-cell transplant. Bold indicates statistical significance.

a Model-based adjustment for medical service to evaluate infectious problem and adjustment for infectious problem to analyze medical service.

Comparison of antifungal and antibiotic PAF recommendation and acceptance rates identified several notable differences (Table 1). Recommendations were more likely to be identified during antifungal PAF than antibiotic PAF for patients located in the CVICU or who were receiving the audited antimicrobial to treat a respiratory tract infection. Antifungal PAF recommendations for skin and soft-tissue infection (SSTI) and urinary tract infection (UTI) were more likely to be accepted than antibiotic PAF recommendations for the same infectious problems. We did not find any significant differences in the acceptance rates of antibiotic and antifungal PAF recommendations when analyzed at the level of medical service. Recommendations for antimicrobial discontinuation or monitoring were more likely during antifungal audits than antibiotic audits, and recommendations to change the antimicrobial agent or modify the duration were more likely for antibiotic compared to antifungal audits (Table 2).

**Table 2.** Comparison of Antifungal and Antibiotic Prospective Audit and Feedback Recommendations

Recommendation Type	Antifungal, No. (%)		Antibiotic, No. (%)		Crude Difference (95% CI)^a^
	Recs (N=379)	Recs Accepted (N=298)	Recs (N=2,467)	Recs Accepted (N=1,982)	
Discontinue	119 (31)	84 (28)	552 (22)	421 (21)	**9 (3.9 to 14.1)**
Optimize regimen	68 (18)	63 (21)	427 (17)	374 (19)	0.6 (−3.7 to 4.9)
IV to PO	45 (12)	25 (8)	236 (10)	173 (9)	2.3 (−1.3 to 5.9)
Monitoring	41 (11)	38 (13)	64 (3)	58 (3)	**8.2 (4.9 to 11.6)**
Clarify	33 (9)	32 (11)	172 (7)	162 (8)	1.7 (−1.4 to 4.9)
Change agent	21 (6)	16 (5)	355 (14)	263 (13)	−**8.8 (**−**11.7 to -6)**
ID consultation	21 (6)	15 (5)	166 (7)	133 (7)	−1.2 (−3.8 to 1.5)
Duration modification	18 (5)	15 (5)	455 (18)	359 (18)	−**13.7 (**−**16.5 to** −**10.9)**
Lengthen duration	0 (0)	0 (0)	4 (0)	4 (0)	NA
Other	13 (3)	10 (3)	36 (1)	35 (2)	1.7 (−0.3 to 3.7)

Note. Recs, recommendations; CI, confidence interval; ID, infectious diseases; NA, not applicable; IV, inteavenous; PO, per oral. Bold indicates statistical significance.

a Based on binomial distribution.

## Discussion

Our study yielded several important findings. First, 21% of antifungal audits at our hospital were associated with a recommendation, highlighting a significant opportunity to improve antifungal prescribing in hospitalized children, particularly those in the CVICU or those receiving antifungals for sepsis or respiratory tract infections. Second, the overall rate of antifungal PAF recommendation acceptance was high and was not significantly different from the rate of antibiotic recommendation acceptance, indicating that prescribers were receptive to this ASP intervention. Third, our analysis revealed several differences in the recommendation and acceptance rates of antifungal and antibiotic PAF for specific infections (eg, UTI and SSTI) and types of recommendations (eg, discontinuation or duration modification). Finally, units with fewer antifungal audits but high recommendation rates (eg, CVICU) may represent significant opportunities for antifungal optimization. Taken together, these findings suggest that antifungal PAF has the potential to identify important patterns of antifungal utilization and may be an effective tool for optimizing antifungal prescribing in the pediatric setting.

Our data are consistent with previously published epidemiologic surveys reporting that the most audited antifungal was fluconazole,^10,11^ most common indication for audited antifungals was prophylaxis,^12^ and most antifungal audits occurred among Heme/Onc/SCT patients. However, the most audited antifungal agents and indications did not necessarily reflect the areas with the highest rate of recommendations and potential for optimizing antifungal prescribing. For example, liposomal amphotericin B, not fluconazole, had the highest rate of recommendation during antifungal PAF. Children’s hospitals with ASPs included in an antifungal stewardship survey reported that the majority performed PAF on liposomal amphotericin B; however, only 40% restricted liposomal amphotericin B to require ID approval or consultation prior to use.^1^ Although PAF may be more effective at decreasing antibiotic use compared to restriction,^13,14^ studies comparing antifungal PAF and restriction are lacking. Future investigations are warranted to evaluate the impact of restricting specific antifungal agents on appropriate prescribing, PAF, and patient outcomes.

Clinical practice guidelines commonly incorporate both diagnostic and therapeutic recommendations. Unfortunately, rapid and accurate diagnostics for invasive fungal disease (IFD) are limited, hindering antifungal stewardship and delaying definitive antifungal therapy.^15^ In our study, antifungal use for sepsis and respiratory tract infection had the highest rate of PAF recommendation, and antifungal recommendations were more common than antibiotic recommendations to optimize respiratory tract infection treatment, possibly reflecting the difficulty in establishing a definitive IFD diagnosis. Challenges related to diagnosing IFD,^15,16^ such as the difficulty in accurately confirming or ruling out the diagnosis, may contribute to the high number of recommendations for patients with sepsis or respiratory tract infection, as well as the high frequency of discontinuation recommendations. The incorporation of sensitive, noninvasive diagnostics, such as fungal cell-free DNA PCR, into clinical practice guidelines could improve the diagnostic process and subsequent antifungal treatment decisions.^16^

Although it may seem intuitive to focus on antifungal stewardship in the Heme/Onc/SCT units given the high volume of prescribing, we found the highest yield of recommendation identification in the CVICU. Given the large volume and highly protocolized use of antifungal prophylaxis, we found PAF in the Heme/Onc/SCT units to be inefficient. In contrast, our CVICU accounted for one-quarter of the antifungal prophylaxis recommendations where an antifungal prophylaxis protocol was lacking, highlighting an opportunity for collaboration and creation of a protocol in units without standardization. Importantly, we would not have identified antifungal prophylaxis for patients in the CVICU as an area of opportunity if we had not first performed antifungal PAF in this unit. Although PAF may be an inefficient strategy of ASP,^9,13^ it remains crucial to leverage PAF data to identify new opportunities to improve antimicrobial use, monitor adherence to existing institutional protocols and guidelines, and prevent blind spots of inappropriate prescribing.

The characteristics of PAF recommendations differed between antifungals and antibiotics. Like previously published data,^1^ the most common antifungal PAF recommendation at our hospital was to discontinue the agent. Our unique comparison revealed that the recommendation for antimicrobial monitoring was more common for antifungals compared to antibiotics; this finding highlights an opportunity to improve antifungal prescribing. The interpatient variability of pharmacokinetic properties and relatively narrow therapeutic index of antifungals distinguishes them from many antibiotics and necessitates appropriate toxicity and therapeutic drug monitoring (TDM), when applicable. Based on our findings, we collaborated with members of the Heme/Onc/SCT and solid-organ transplant teams to develop an azole TDM guideline to standardize recommendations and possibly reduce PAF recommendations around monitoring. An investigation of the impact of this guidance on antifungal PAF is merited.

This study had several limitations. It was conducted at a single center and may not reflect the practice of PAF and antifungal prescribing at other institutions. Complementary stewardship efforts at our hospital, including formulary restrictions and clinical guidelines, are likely to have influenced our findings. Like previously published studies, we were unable to discern the impact of this intervention on clinical outcomes, including mortality and hospital length of stay. However, the significant proportion of audits with a recommendation suggests an important opportunity for antifungal optimization using a PAF strategy.

Implementation of antifungal PAF may be effective in promoting appropriate use of antifungal agents, particularly in areas of the hospital where utilization practices are not standardized. However, to ensure the successful implementation of antifungal PAF, tailored approaches that align with institutional prescribing practices and integration of complementary stewardship strategies are crucial. Given the potential impact of inappropriate antifungal use on patient outcomes and healthcare costs, further investigations are needed to evaluate clinical outcomes associated with antifungal PAF and to determine the optimal approach for antifungal stewardship.

## Supplementary material

For supplementary material accompanying this paper visit https://doi.org/10.1017/ice.2023.129. click here to view supplementary material
