# Supplementary material for: Antifungal stewardship in practice: Insights from a prospective audit and feedback program
Source: Infect Control Hosp Epidemiol. 2023 Jun 29;44(12):2017–21. doi: 10.1017/ice.2023.129 (PMC10755142; doi:10.1017/ice.2023.129)
Supplement: Supplementary file 1 [file S0899823X23001290sup001.pdf]

SUPPLEMENTARY MATERIAL

Table 1. Categorization of Infectious Problems

**Group**	**Infectious Problems**
Bloodstream infection	Bacteremia
	Central-line associated blood stream infection
	Fungemia
Febrile neutropenia	Febrile neutropenia without identified source of infection
Gastrointestinal/IAI	Appendicitis
	Esophageal candidiasis
	Intra-abdominal process
	Clostridiodes difficile infection
	Necrotizing enterocolitis
Head and neck	Dental abscess
	Mastoiditis
	Oral candidiasis
	Orbital and periorbital cellulitis
	Oropharyngeal infection
	Otitis media
	Retropharyngeal abscess
	Sinusitis
Non-infectious	Non-infectious
Other	Chorioretinitis
	Endocarditis
	Lymphadenitis
	Mediastinitis

	Meningitis
	None identified
	Osteomyelitis
	Septic arthritis
	Sexually transmitted infection
	Ventriculoperitoneal shunt infection
Prophylaxis	Perioperative prophylaxis
	Prophylaxis
Respiratory infection	Acute chest syndrome
	Aspiration pneumonia
	Community acquired pneumonia
	Cystic fibrosis exacerbation
	Pneumonia
	Presumed respiratory infection
	Tracheitis
	Ventilator-associated pneumonia/hospital-associated pneumonia
Sepsis	Culture negative sepsis
	Suspected fungal infection
	Rule-out central-line associated blood stream infection
	Rule-out sepsis
	Sepsis/septic shock
Skin and soft tissue infection	Animal bite
	Omphalitis
	Skin and soft tissue infection
	Surgical site infection

Urinary tract infection	Catheter-associated urinary tract infection
	Urinary tract infection

Table 2. Medical Service Categorization

Medical Service Group	Medical Services
Cardiovascular Intensive Care	Cardiovascular Intensive Care
Hematology/Oncology/Stem Cell	Hematology/Oncology
	Stem Cell Transplant
Intensive Care Unit	Intensive Care
Medical services	Adolescent Medicine
	Cardiac Cath
	Cardiology
	Dentistry
	Dermatology
	Endocrinology
	Gastroenterology
	General Pediatrics
	Immunology & Allergy
	Nephrology
	Neurology
	Pain Management
	Pulmonary
	Rheumatology
Neonatology	Neonatology
Solid Organ Transplant	Cardiovascular Transplant
	Kidney Transplant
	Liver Transplant
	Transplant Surgery

Surgical services	Cardiovascular Surgery
	General Surgery
	Hand
	Neurosurgery
	Ophthalmology
	Orthopedic Surgery
	Otolaryngology (ENT)
	Plastic Surgery
	Urology

Table 3. Categorization of Recommendation Type

Recommendation Type Group	Recommendation Type
Change agent	Broaden empirically
	Broaden based on culture/sensitivity
	Change antimicrobial (availability)
	Change antimicrobial based on culture/sensitivity
	Narrow based on culture/sensitivity
	Narrow empirically
Lengthen duration	Lengthen duration
Clarify	Clarify indication/plan
IV to PO	IV to PO
Monitoring	Monitoring
Optimize dose regimen	Decrease dose
	Decrease frequency
	Increase dose
	Increase frequency
	Prolonged infusion
PO to IV	PO to IV
Duration modification	Duration suggestion
	Shorten duration
Discontinue	Stop (Consolidate to fewer agents)
	Stop (No indication)
	Stop (Redundant therapy)
ID consult	ID consult
Other	Other
